# Effectiveness of the acute stroke care map program in reducing in-hospital delay for acute ischemic stroke in a Chinese urban area: an interrupted time series analysis

**DOI:** 10.3389/fneur.2024.1364952

**Published:** 2024-04-18

**Authors:** Rui Wen, Miaoran Wang, Wei Bian, Haoyue Zhu, Ying Xiao, Jing Zeng, Qian He, Yu Wang, Xiaoqing Liu, Yangdi Shi, Zhe Hong, Bing Xu

**Affiliations:** ^1^Shenyang Tenth People’s Hospital, Shenyang, China; ^2^Affiliated Central Hospital of Shenyang Medical College, Shenyang Medical College, Shenyang, China; ^3^Shenyang First People’s Hospital, Shenyang Medical College, Shenyang, China; ^4^ChongQing Medical University, ChongQing, China

**Keywords:** acute stroke care map, in-hospital delay, interrupted time-series analysis, acute ischemic stroke, program

## Abstract

**Background:**

Timely intravenous thrombolysis (IVT) is crucial for improving outcomes in acute ischemic stroke (AIS) patients. This study evaluates the effectiveness of the Acute Stroke Care Map (ASCaM) initiative in Shenyang, aimed at reducing door-to-needle times (DNT) and thus improving the timeliness of care for AIS patients.

**Methods:**

An retrospective cohort study was conducted from April 2019 to December 2021 in 30 hospitals participating in the ASCaM initiative in Shenyang. The ASCaM bundle included strategies such as EMS prenotification, rapid stroke triage, on-call stroke neurologists, immediate neuroimaging interpretation, and the innovative Pre-hospital Emergency Call and Location Identification feature. An interrupted time series analysis (ITSA) was used to assess the impact of ASCaM on DNT, comparing 9 months pre-intervention with 24 months post-intervention.

**Results:**

Data from 9,680 IVT-treated ischemic stroke patients were analyzed, including 2,401 in the pre-intervention phase and 7,279 post-intervention. The ITSA revealed a significant reduction in monthly DNT by −1.12 min and a level change of −5.727 min post-ASCaM implementation.

**Conclusion:**

The ASCaM initiative significantly reduced in-hospital delays for AIS patients, demonstrating its effectiveness as a comprehensive stroke care improvement strategy in urban settings. These findings highlight the potential of coordinated care interventions to enhance timely access to reperfusion therapies and overall stroke prognosis.

## Introduction

Stroke remains a leading cause of disability worldwide, with nearly two-thirds of survivors experiencing long-term impairments (1). Strokes are categorized into ischemic, which make up 85% of cases, and hemorrhagic strokes, accounting for the remaining 15%. Prompt diagnostic evaluation is essential across all types of strokes to confirm the diagnosis and initiate the appropriate treatment swiftly. While direct treatment for hemorrhagic strokes often faces challenges due to the complexity of addressing the underlying cause, timely intervention in ischemic stroke management is crucial to prevent rapid progression to irreversible brain damage (2).

Shorter door-to-needle times (DNT) are crucial for enhancing the prognosis of acute ischemic stroke (AIS) patients (3), not only improving in-hospital outcomes but also contributing to better long-term recovery and reduced disability. The Target: Stroke initiative, launched in January 2010 by the American Heart Association and the American Stroke Association, aimed to help hospitals achieve shorter DNTs. Through the distribution of a series of best practice strategies, the initiative set targets to reduce DNT to within 60 min for at least 50% of patients receiving tissue plasminogen activator (tPA), subsequently raising this goal to 75% of patients, with an added objective of achieving DNT within 45 min for at least 50% of treated patients (4, 5).

The burden of stroke in China has significantly increased, with stroke being the leading cause of death. The prevalence, incidence, and mortality rates of stroke in China in 2020 were 2.6%, 505.2 per 100,000 person-years, and 343.4 per 100,000 person-years, respectively. It was estimated that there were 3.4 million incident cases, 17.8 million prevalent cases, and 2.3 million deaths from stroke among the Chinese population aged 40 years and older in 2020. Ischemic stroke constituted 86.8% of all incident strokes, highlighting the urgent need for an improved stroke prevention strategy in the general Chinese population (6, 7).

In alignment with national efforts, supported by the Stroke Prevention Project Committee of the Chinese National Health Commission (SPPCCNHC), various cities, including Shenzhen, Yantai, Hangzhou, Suzhou, Qingdao, and Shenyang, have launched the Acute Stroke Care Maps (ASCaMs). Contrary to the initial impression that may be formed by the term, ASCaMs are not merely geographical or digital mapping tools but represent a comprehensive intervention program. This initiative integrates prehospital emergency medical services (EMS) and in-hospital strategies to streamline the entire care pathway for AIS patients. It seeks to identify eligible hospitals and aims primarily at minimizing in-hospital delays while ensuring the delivery of high-quality thrombolytic and endovascular services. The ASCaM strategy encapsulates a bundle of improvements, including EMS prenotification, emergency department protocol optimization, constant availability of stroke specialists, efficient stroke triage and alert systems, prompt neuroimaging analysis and the innovative Pre-hospital Emergency Call and Location Identification feature. The metaphorical use of “map” in ASCaM signifies a structured guide to navigate the urgent care required for stroke patients effectively, enhancing the overall stroke care system rather than suggesting a literal navigational tool.

While the implementation of such comprehensive strategies has been shown to reduce in-hospital delays, enhance thrombolysis and endovascular thrombectomy rates, and improve clinical outcomes for AIS patients, the specific impact of tailored improvement strategies within the densely populated and complex stroke care systems of China has yet to be fully explored. This study aims to evaluate the citywide implementation of ASCaM in Shenyang, focusing on its efficacy in reducing DNT and thereby improving in-hospital delays, utilizing an interrupted time series analysis as a robust method for assessing the longitudinal impact of health interventions.

## Materials and methods

### Study design and data sources

**Figure 1:**
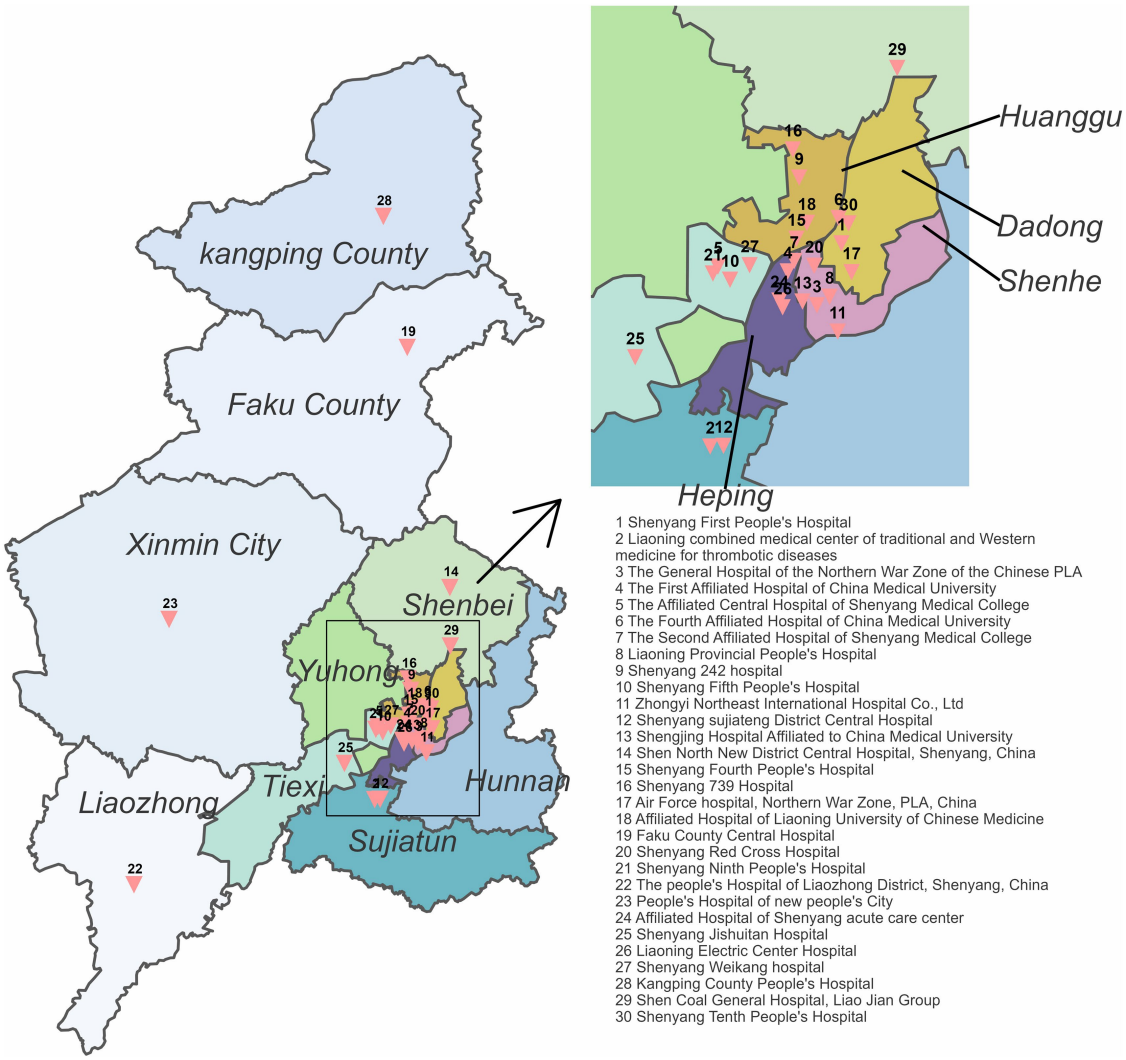
Shenyang acute stroke care map. The geographical locations of all 30 participating hospitals are displayed.

This retrospective cohort study, approved by the Research Ethics Committee of Shenyang First’s People Hospital (Number: 2023SYKYPZ09), evaluated the impact of the Shenyang Acute Stroke Care Map (ASCaM) on reducing door-to-needle times (DNT) for ischemic stroke patients undergoing intravenous thrombolysis (IVT). Data for this study were collected from 30 hospitals across Shenyang, as detailed in Figure 1, highlighting the geographical distribution and inclusion of these facilities. ASCaM, which includes comprehensive interventions such as EMS prenotification, rapid stroke triage, the availability of on-call stroke neurologists, and immediate neuroimaging interpretation, was rolled out across selected hospitals in Shenyang on January 1, 2020. The study period spanned from April 2019 to December 2021, segmented into a 9-month pre-intervention phase (April 2019 to December 2019) and a 24-month post-intervention phase (January 2020 to December 2021), to compare the effectiveness of ASCaM in streamlining acute stroke care. The evaluation focused on median DNT as a measure of in-hospital delay performance.

To accurately assess ASCaM’s influence, we employed an interrupted time series (ITS) analysis, examining the trends and levels of DNT before and after the implementation of ASCaM. This methodological approach aims to determine whether the introduction of ASCaM led to significant improvements in the timeliness of stroke care by comparing the period before and after its implementation.

### Intervention description

Acute Stroke Care Map encompasses a comprehensive suite of strategies designed to streamline and enhance stroke care delivery. These strategies include EMS prenotification, advanced emergency department readiness, the constant availability of on-call stroke neurologists, rapid stroke triage systems, immediate interpretation of neuroimaging, and the innovative Pre-hospital Emergency Call and Location Identification feature. This latter addition allows individuals to access the Stroke Care Map via QR code or WeChat for real-time information on nearby hospitals equipped for stroke emergencies, providing details such as hospital locations, stroke center certifications, and emergency capabilities (thrombolysis, intervention). It also includes a self-assessment template for early symptom recognition, advises on making emergency calls, or locating the nearest hospital with navigation support. These interventions, detailed in Table 1, were implemented to significantly enhance the efficiency and responsiveness of acute stroke treatment across the participating hospitals.

**Table 1:** Strategies implemented among Shenyang ASCaM hospitals (with selected references).

Strategies	Description	References
EMS prenotification	Emergency medical personnel inform the hospital’s stroke neurologists in advance about the patient’s medical history and any observed abnormalities.	(4, 5, 8–10)
Readiness of Emergency Department (ED)	The ED is prepared ahead of time with necessary equipment such as intravenous lines, catheters, and monitors for electrocardiography, ensuring readiness for diverse medical needs.	(8)
Constant availability of stroke specialists	Stroke experts, including fellows and neurology residents, are always present in the ED. Neurointerventionists are also available as needed.	(8, 11)
Efficient stroke triage and alert system	A swift and effective triage system for stroke patients is in place, ensuring prompt notification of the specialized stroke team.	(4, 5, 8)
Continuous patient supervision	Patients who are candidates for thrombolysis are constantly supervised by ED staff, typically by stroke nurses, from arrival until the administration of intravenous tPA.	(8)
On-the-spot neuroimaging analysis	Brain scans are immediately analyzed by ED neurologists as soon as they are available.	(4, 5, 8, 12)
Initial decision by neurologists	The decision to proceed with thrombolysis is initially made by the attending neurologists and is subsequently confirmed by a stroke fellow either in person or via phone.	(5, 8)
Prioritization of thrombolysis patients	Patients indicated for thrombolysis receive top priority in the hospital, especially regarding access to neuroimaging and laboratory services.	(8, 13)
Ready access to stroke toolkits	Essential stroke toolkits, which include assessment scales, consent forms, and tPA, are readily available around the clock in the ED.	(4, 5, 8)
Proximity of labs and imaging facilities	The lab and imaging facilities are strategically located as close to the ED as possible, ideally within a very short distance for quick access.	(5, 8, 10, 12, 14)
Pre-hospital emergency call and location identification	Enables access to the Stroke Care Map via QR code or WeChat for real-time information on nearby hospitals equipped for stroke emergencies. Provides hospital locations, stroke center certifications, and emergency capabilities (thrombolysis, intervention). Includes a self-assessment template for early symptom recognition and advises on emergency calls or locating the nearest hospital with navigation support.	

EMS, Emergency medical services; ED, Emergency department; and tPA, Tissue plasminogen activator.

### Interrupted time series analysis

Following the guidelines outlined by Bernal et al. (15), our ITS analysis was designed to assess alterations in the level and trend of monthly thrombolysis rates before and after the initiation of ASCaM, with January 2020 marked as the pivotal breakpoint. This approach allowed us to closely monitor changes attributable to the intervention across the specified timeframe.

To ensure the robustness of our time series data against potential seasonal effects, we employed the Ljung-Box test. The Durbin-Watson statistic further aided in verifying the independence of observations, addressing any concerns regarding autocorrelation within the dataset.

To deepen our understanding of ASCaM’s impact, we conducted subset analyses segmented by age groups (<65 and ≥ 65 years) and NIHSS score categories (<6 and ≥ 6 points). These analyses aimed to discern the intervention’s differential effects across various demographic and clinical presentations.

Our regression model incorporated multiple parameters to elucidate the dynamics of DNT changes throughout the study period. These included: (i) time elapsed since the study start (T, 1–33, representing the total months); (ii) T_i_, the time point when ASCaM begins (X_t_ = 1 for T ≥ T_i_); (iii) a dummy variable for the intervention phase (X_t_, 0 for pre-intervention, 1 for post-intervention); and (iv) ε, the error term. The model focused on short-term level changes, not long-term trend changes. The segmented regression model was as follows:


$$Y_{t}=\beta_{0}+\beta_{1}T+\beta_{2}X_{t}+\beta_{3}\left(T-T_{i}\right)\cdot X_{t}+\varepsilon_{t}$$

where *Y_t_* represents the median pre-hospital delay time in a month, *t* ranges from 1 to 33 months, *β_0_* is the baseline intercept, *β_1_* is the average monthly change in median DNT before ASCaM implementation, *β_2_* is the immediate effect of ASCaM implementation, *β_3_* is the change in the slope of the monthly median DNT following ASCaM implementation, and *ε_t_* is the error term.

### Statistical analyses

Descriptive statistics were employed to summarize patient demographics and clinical characteristics. Categorical variables were expressed as *n* (%), while continuous variables were reported as mean (SD) or median (interquartile range). Differences in baseline characteristics between groups were analyzed using independent sample *t*-tests, Mann–Whitney U tests for continuous variables, and the chi-squared test or Fisher’s exact test for categorical variables, as appropriate.

All statistical tests were two-sided, and a *p* value <0.05 was considered statistically significant. All statistical analyses were conducted using R v4.0.1.^1^

## Results

**Figure 2:**
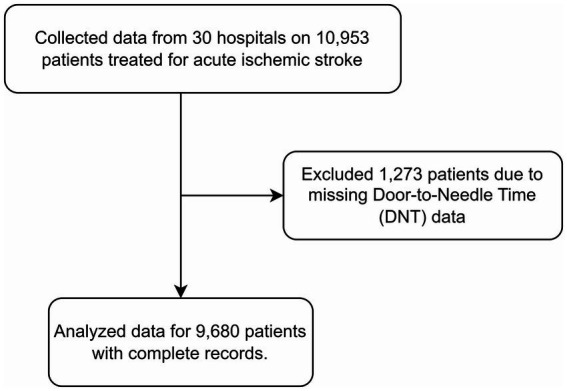
Patient selection process.

The final study cohort, as depicted in Figure 2, comprised a total of 9,680 ischemic stroke patients—2,401 in the pre-intervention period and 7,279 following the implementation of the ASCaM intervention. Demographic and clinical characteristics of ischemic stroke patients treated with intravenous tPA before and after the intervention are summarized in Table 2.

**Table 2:** Demographic and clinical characteristics of included ischemic stroke patients, before and after the introduction of the ASCaM intervention.

Characteristic	Total (*n* = 9,680)	Intervention^1^		*p*
		Before (*n* = 2,401)	After (*n* = 7,279)	
Age, Mean ± SD	64.87 ± 11.28	64.54 ± 11.52	64.98 ± 11.2	0.106
Gender, *n* (%)				0.729
Male	6,766 (70)	1,686 (70)	5,080 (70)	
Female	2,911 (30)	715 (30)	2,196 (30)	
Admission NIHSS score, Mean ± SD	7.85 ± 6.56	8 ± 6.52	7.8 ± 6.57	0.205
BMI, Mean ± SD	24.25 ± 3.64	24.26 ± 3.6	24.25 ± 3.66	0.887
Blood pressure, Mean ± SD, mmHg	154.48 ± 21.15	155.14 ± 21.94	154.26 ± 20.89	0.085
DNT, Median (Q1,Q3)	45 (30, 63)	50 (35, 74)	43 (30, 60)	<0.001

BMI, Body mass index; DNT, Door-to-needle time. ^1^ASCaM implementation.

The baseline demographic profile did not differ significantly between the two groups. There were no statistically significant differences when comparing the baseline characteristics of those with and without the implementation of the ASCaM intervention. However, a statistically significant reduction in DNT was observed before and after the intervention (median changed from 50 to 43, *p* < 0.001).

The ITS analysis showed no seasonality, as indicated by the *F*-test (*p* = 0.373). The Durbin-Watson statistic was close to 2.0 (Durbin-Watson = 1.776), indicating no autocorrelation in the dataset.

**Figure 3:**
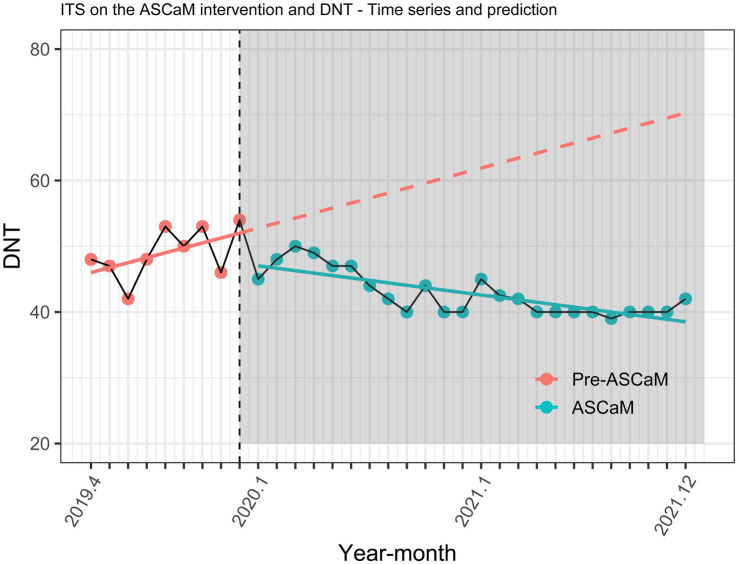
Interrupted time series analysis, all ischaemic stroke patients.

The effect of the ASCaM on DNT trends is illustrated in Figure 3 and Table 3. A statistically significant change in the level of DNT by −5.727 minutes was observed (95% confidence interval [CI] −9.888 to −1.566, *p* = 0.012). Furthermore, a monthly decrease in DNT of −1.12 minutes post-ASCaM implementation was noted (95% CI −1.786 to −0.454).

**Table 3:** Results of the interrupted time series analysis of the impact of the ASCaM intervention on DNT: existing trend, changes in trend, and changes in level after the ASCaM implementation (*n* = 9,680).

	Time trend (monthly)		Intervention-Slope change		Intervention-Intercept change	
	*B* (95% CI)^*^	*p*	*B* (95% CI)^*^	*p*	*B* (95% CI)^*^	*p*
Full dataset	0.75 (0.101;1.399)	0.031	−1.12 (−1.786;-0.454)	0.003	−5.727 (−9.888;-1.566)	0.012
Age < 65	0.883 (0.07;1.696)	0.042	−1.187 (−2.02;-0.354)	0.009	−6.695 (−11.903;-1.487)	0.018
Age ≥ 65分	0.7 (−0.164;1.564)	0.123	−1.089 (−1.975;-0.203)	0.022	−6.613 (−12.146;-1.08)	0.026
NIHSS ≥ 6分	0.233 (−0.584;1.05)	0.58	−0.746 (−1.585;0.093)	0.092	−2.494 (−7.731;2.743)	0.358
NIHSS <6分	1.317 (0.345;2.289)	0.013	−1.522 (−2.52;-0.524)	0.006	−10.677 (−16.91;-4.444)	0.002

**Figure 4:**
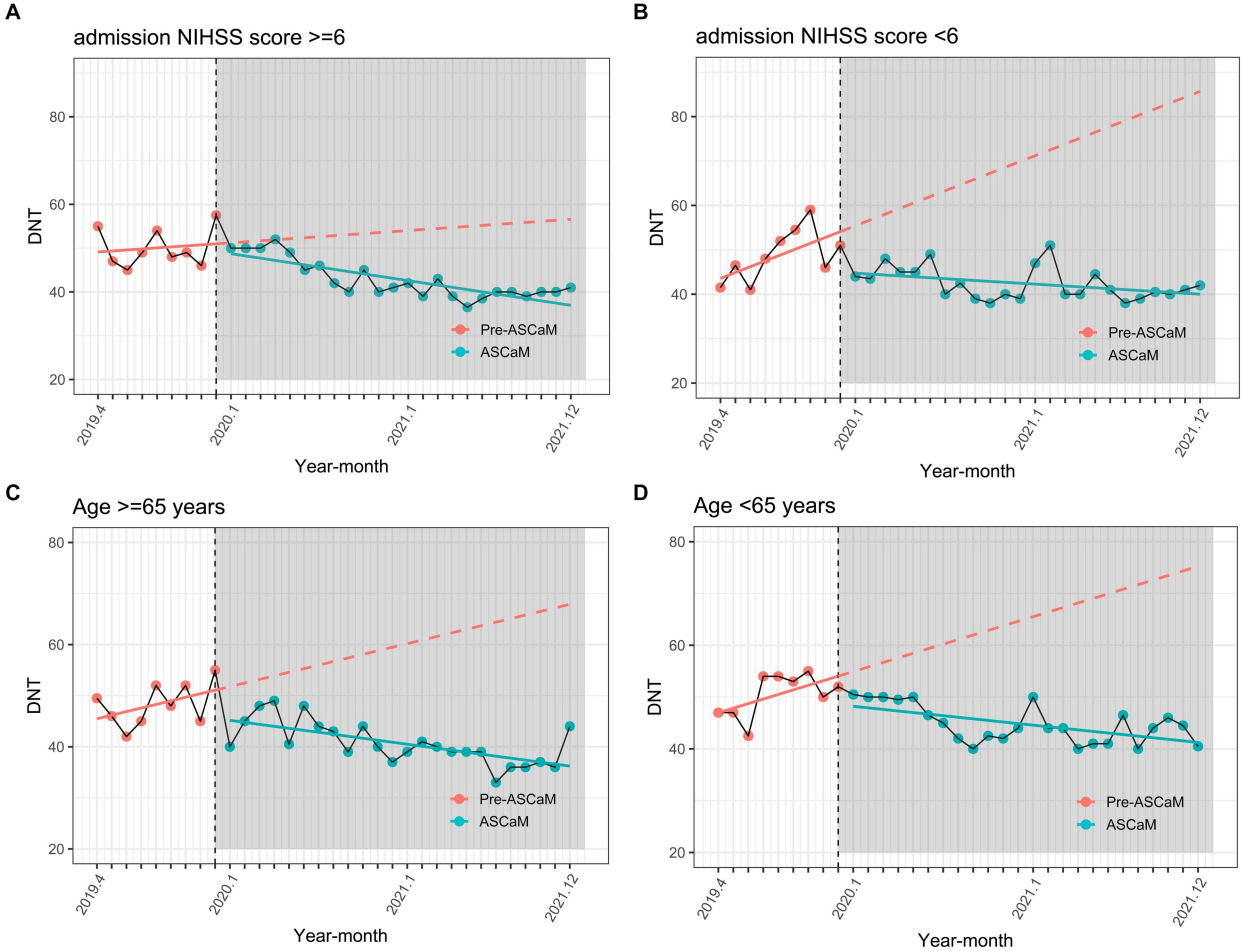
Subset interrupted time series analysis using admission NIHSS score and age as stratifying variables. **(A)** Admission NIHSS score ≥6; **(B)** Admission NIHSS score <6; **(C)** Age ≥65 years; **(D)** Age <65 years.

However, the pre-intervention upward trend must be considered (0.75 min, 95% CI 0.101–1.399, *p* < 0.001), resulting in an overall descending trend, with *a* − 0.37 min monthly decrease in DNT post-intervention (Table 3). Subset analyses (Figure 4; Table 3) showed that the effect of ASCaM in reducing DNT was statistically significant in patients with an NIHSS <6 and in the group aged below 65 years, but no significant trend was identified for patients with an NIHSS ≥6, and the group aged 65 years or older.. Full results of the ITS analyses are available in Table 2.

## Discussion

This study assessed the performance of in-hospital delay, measured by DNT, following the implementation of ASCaM in Shenyang. The findings reveal that ASCaM appears to have had a positive effect on decreasing in-hospital delay, providing evidence of its effectiveness in improving time to reperfusion therapy during the acute phase. A previous study reported that the implementation of a regional Acute Stroke Care Map increased thrombolysis rates for acute ischemic stroke in a Chinese urban area within just 3 months (8). This study supports our findings, as lower DNT implies that AIS patients have more opportunities to receive intravenous thrombolysis. However, the previous study did not demonstrate the long-term effectiveness of ASCaM. Our study expands upon this research by examining the related DNT trends from 2019 to 2021, during which ASCaM was implemented.

Despite the modest decrease in DNT observed in our study, it is important to emphasize the clinical significance of even small reductions in treatment time for AIS. Literature consistently demonstrates that the efficacy of IVT in AIS is highly time-dependent, with earlier treatment associated with better outcomes (16–21). This underscores the relevance of our findings, as even a reduction of a few minutes can be critical in the context of stroke care where “time is brain” (22, 23).

The main finding of this study was the significant decrease in the trend of DNT following the implementation of ASCaM in January 2020. The pattern of increasing trends immediately after the intervention may be attributed to the lockdown that began when Shenyang declared states of emergency in early February. The lockdown had a tremendous effect on mobility, causing DNT to increase because patients had to strictly adhere to the hospital’s epidemic prevention regulations when accessing medical facilities. As the optimization of epidemic prevention policies was implemented, DNT showed a slight decreasing trend. In general, while the epidemic may have temporarily increased DNT, the overall trend remained downward following the implementation of ASCaM.

To further elucidate the aspects of our intervention bundle that we believe were instrumental in this achievement, we identified several key strategies. These include: EMS Prenotification and Streamlined ED Protocols, ensuring that the hospital team is prepared in advance for the incoming stroke patient, which minimizes delays in initial assessment and treatment initiation. The Constant Availability of Stroke Specialists and an Efficient Stroke Triage and Alert System in the ED, facilitating swift decision-making and reducing time to thrombolysis. The innovative Pre-hospital Emergency Call and Location Identification feature through the “Stroke Care Map,” which aids in reducing the time from symptom onset to hospital arrival by guiding patients to the nearest stroke-ready hospital. On-the-Spot Neuroimaging Analysis, enabling rapid diagnosis and thus, faster initiation of treatment. These elements combined addressed various stages of the stroke care pathway, synergistically reducing both pre-hospital and in-hospital delays.

Although a decrease in DNT was observed following the implementation of ASCaM across the entire dataset, no significant trends were identified among patients with an NIHSS ≥6 and those aged ≥65 years. One possible explanation for this finding is that patients with an NIHSS ≥6 may struggle to provide timely feedback to emergency personnel due to their severe condition, resulting in inaccurate information transmission between patients and ASCaM hospitals and extended handover times. Furthermore, patients aged ≥65 years may experience verbal errors, forgetfulness, and slow thinking, which could reduce both the rate and quality of information transmission, ultimately increasing the preparation time required before receiving treatment.

The research results suggest that the benefits of the Shenyang Stroke Emergency Map Network are not as pronounced for individuals aged 65 and older or those with higher NIHSS scores. Therefore, it is crucial to pay more attention to these populations and develop tailored measures specifically for them, such as employing specialized and experienced medical staff to triage these patients.

Intravenous thrombolysis (IVT) notably enhances clinical outcomes for eligible patients with acute ischemic stroke (AIS), its efficacy, however, is highly time-sensitive (24). Diminishing in-hospital delay time is of paramount importance for AIS patients; it can heighten access to acute reperfusion therapies and decrease overall mortality (25).

Prior to the citywide implementation of ASCaM in Shenyang, the landscape of stroke care varied significantly across different hospitals. While the national median DNT for stroke care in China has been reported to be between 80 and 100 min (26), indicating a considerable delay in initiating thrombolytic therapy, some hospitals in Shenyang had already begun to implement changes aimed at improving stroke care efficiency. These early adopters of stroke care optimization strategies may have contributed to a baseline DNT that was notably lower than the national average, reflecting a proactive approach to enhancing stroke response times within the region.

Despite these initial efforts, the implementation of ASCaM represented a comprehensive and standardized approach to further reduce in-hospital delays for acute ischemic stroke treatment. The program brought together a bundle of interventions, including but not limited to EMS prenotification, streamlined emergency department protocols, and the availability of stroke specialists, which were variably present in some form at certain hospitals prior to ASCaM’s citywide rollout. The ASCaM initiative aimed to elevate these practices to a new level of efficiency and consistency across all participating hospitals.

Our study observed a notable reduction in DNT following the ASCaM intervention’s implementation, with a median DNT reduced from 50 to 43 min. Interventions that provide advanced notification to hospital stroke neurologists about patients’ medical history and abnormalities by ambulance staff have proven effective in reducing DNT (4, 5, 9). These findings align with earlier studies demonstrating that process enhancements can decrease DNT (3, 27). Nonetheless, our study discovered that the ASCaM intervention’s effect was particularly evident in patients with an NIHSS score of less than 6 and in those below 65 years old. This indicates that we need additional efforts to decrease DNT in elderly patients and those with severe strokes. Customized approaches targeting these groups, which may include specialized triage and care by seasoned medical staff, can ensure that all patients reap the benefits of the ASCaM system’s reduced in-hospital delays.

However, our study demonstrated that the implementation of ASCaM not only decreased DNT but also showed a trend toward further reduction. Furthermore, our research benefits from a large sample size drawn from multi-center registries, which provides significant power to detect the intervention’s effect. The initiation of citywide ASCaMs is a national initiative, and so far, 30 cities have implemented the ASCaM system (28). Shenyang is the sixth city to do so. Yet, there is scant city-based data on the association between ASCaM implementation and improved in-hospital delays. Our study utilized data from 30 hospitals in Shenyang to ascertain that the citywide ASCaM is effective in reducing DNT and improving in-hospital delay. It offers robust support for the implementation of ASCaM in other Chinese cities.

This study acknowledges several limitations. First, while interrupted time series analysis is considered one of the most robust quasi-experimental research designs, especially when a randomized trial is unfeasible or unethical (29, 30), it carries certain limitations. In our investigation of the Acute Stroke Care Map (ASCaM) and its impact on reducing door-to-needle time (DNT) for acute ischemic stroke, we recognize several limitations, particularly stemming from the quasi-experimental design of our study. The use of interrupted time series analysis (ITSA) provides valuable insights from observational data, but it does not offer the same level of control as randomized controlled trials. This design inherently assumes a direct causal relationship between the implementation of ASCaM and the observed changes in DNT. While our data, covering 33 months before and after the implementation, is extensive, it cannot completely eliminate the possibility of external factors or changes in clinical practices over time that might have influenced the outcomes.

Moreover, quasi-experimental designs like ours are often challenged by issues such as the inability to control for all potential confounding variables. Although we have made efforts to account for known confounders, there is always the possibility of unmeasured variables that could affect the results. Additionally, such designs can be susceptible to threats to internal validity, such as maturation effects where changes over time might occur independently of the intervention.

These aspects underscore the need for cautious interpretation of our findings. While the results suggest a beneficial trend in DNT reduction following the implementation of ASCaM, the limitations of our study design imply that these findings should be viewed as preliminary. They highlight the necessity for further research, ideally incorporating more controlled experimental designs, to validate and build upon our results. Our study contributes to the broader discourse on optimizing in-hospital stroke care but also emphasizes the complexities involved in evaluating healthcare interventions in real-world settings.

## Conclusion

This study has conclusively shown that the implementation of the Acute Stroke Care Map (ASCaM) has been instrumental in significantly reducing in-hospital delays for patients experiencing acute ischemic stroke (AIS) in Shenyang. ASCaM stands out as an effective and comprehensive strategy for enhancing the quality of stroke care in densely populated urban environments. By facilitating more timely access to essential reperfusion therapies, ASCaM not only improves immediate treatment outcomes but also has a promising impact on the broader prognosis of stroke patients. Our findings advocate for the broader adoption of similar coordinated care interventions across urban centers in China, aiming to elevate the standard of stroke care and patient outcomes nationwide.

## Data availability statement

The raw data supporting the conclusions of this article will be made available by the authors, without undue reservation.

## Ethics statement

The studies involving humans were approved by the Research Ethics Committee of Shenyang First’s People Hospital. The studies were conducted in accordance with the local legislation and institutional requirements. Written informed consent for participation was not required from the participants or the participants’ legal guardians/next of kin in accordance with the national legislation and institutional requirements.

## Author contributions

RW: Writing – original draft. MW: Writing – review & editing. WB: Writing – review & editing. HZ: Writing – review & editing. YX: Writing – review & editing. QH: Writing – review & editing. YW: Writing – review & editing. XL: Writing – review & editing. YS: Writing – review & editing. ZH: Writing – review & editing. BX: Project administration, Writing – review & editing. JZ: Writing – review & editing.

## Footnotes

^1^ http://www.R-project.org
